# Current Topics of Relevance to the Xenotransplantation of Free Pig Islets

**DOI:** 10.3389/fimmu.2022.854883

**Published:** 2022-04-01

**Authors:** Lisha Mou, Guanghan Shi, David K.C. Cooper, Ying Lu, Jiao Chen, Shufang Zhu, Jing Deng, Yuanyuan Huang, Yong Ni, Yongqiang Zhan, Zhiming Cai, Zuhui Pu

**Affiliations:** ^1^ Department of Hepatopancreatobiliary Surgery, Shenzhen Institute of Translational Medicine, Health Science Center, The First Affiliated Hospital of Shenzhen University, Shenzhen Second People’s Hospital, Shenzhen, China; ^2^ Shenzhen Xenotransplantation Medical Engineering Research and Development Center, Shenzhen Institute of Translational Medicine, Health Science Center, The First Affiliated Hospital of Shenzhen University, Shenzhen Second People’s Hospital, Shenzhen, China; ^3^ Faculty of Arts and Science, University of Toronto, Toronto, ON, Canada; ^4^ Center for Transplantation Sciences, Department of Surgery, Massachusetts General Hospital, Boston, MA, United States; ^5^ Department of Life Science, Bellevue College, Bellevue, WA, United States; ^6^ Imaging Department, Shenzhen Institute of Translational Medicine, The First Affiliated Hospital of Shenzhen University, Shenzhen Second People’s Hospital, Shenzhen, China

**Keywords:** immunosuppression, islets, nonhuman primate, pig, genetically-engineered, type 1 diabetes, islet transplantation, xenotransplantation

## Abstract

Pig islet xenotransplantation is a potential treatment for patients with type 1 diabetes. Current efforts are focused on identifying the optimal pig islet source and overcoming the immunological barrier. The optimal age of the pig donors remains controversial since both adult and neonatal pig islets have advantages. Isolation of adult islets using GMP grade collagenase has significantly improved the quantity and quality of adult islets, but neonatal islets can be isolated at a much lower cost. Certain culture media and coculture with mesenchymal stromal cells facilitate neonatal islet maturation and function. Genetic modification in pigs affords a promising strategy to prevent rejection. Deletion of expression of the three known carbohydrate xenoantigens (Gal, Neu5Gc, Sda) will certainly be beneficial in pig organ transplantation in humans, but this is not yet proven in islet transplantation, though the challenge of the ‘4th xenoantigen’ may prove problematic in nonhuman primate models. Blockade of the CD40/CD154 costimulation pathway leads to long-term islet graft survival (of up to 965 days). Anti-CD40mAbs have already been applied in phase II clinical trials of islet *allo*transplantation. Fc region-modified anti-CD154mAbs successfully prevent the thrombotic complications reported previously. In this review, we discuss (I) the optimal age of the islet-source pig, (ii) progress in genetic modification of pigs, (iii) the immunosuppressive regimen for pig islet xenotransplantation, and (iv) the reduction in the instant blood-mediated inflammatory reaction.

## Introduction

Type 1 diabetes (T1D) is a chronic autoimmune disease characterized by pancreatic islet cell destruction by CD4^+^ and CD8^+^ T cells and autoantibodies, resulting in insulin deficiency and hyperglycemia (1). Conventional treatment of T1D includes exogenous insulin therapy, which reduces, but may not prevent, the development of the long-term complications of hyperglycemia. In late-stage T1D patients, especially those with ‘brittle’ diabetes, it is difficult to prevent complications such as cardiovascular disease, retinopathy, nephropathy, and life-threatening hypoglycemic episodes (1).

Islet *allo*transplantation has been identified as an efficient therapy for T1D, but, faced with the shortage of pancreases from deceased human donors, pig-to-human islet xenotransplantation has emerged as a potential alternative (2). Although pig-to-nonhuman primate (NHP) islet xenotransplantation has resulted in insulin independence, several problems remain.

The age of the islet-source pig may be important to islet quality. Adult pigs have a mature islet structure, lower galactose-α1,3-galactose (Gal) expression on islets, and a higher islet yield (3). Neonatal pig islets are easier to isolate and at a lower cost (3). To overcome immunological rejection of pig-to-NHP islet transplants, genetic modification of the source pig plays an important role by deleting xenoantigen expression and introducing human ‘protective’ proteins (4). New alternative modifications, e.g., expression of programmed cell death ligand 1 (PD-L1), are being explored. A consensus has been reached that, in regard to the transplantation of pig organs into humans, the expression of the three known carbohydrate xenoantigens (Gal, Neu5Gc, Sda) should be deleted (resulting in triple-knockout [TKO] pigs) (4, 5), but this remains uncertain after pig islet transplantation. However, there is a limitation in the TKO pig-to-NHP model because of the problem of the ‘4th xenoantigen’.

The selection of the immunosuppressive regimen plays a critical role in preventing the adaptive immune response (6). Although conventional immunosuppressive regimens are inefficient in preventing the adaptive response to pig cells, blockade of the CD40/CD154 costimulation pathway is successful, and has resulted in insulin-independence for a maximum of 965 days) (7). Emerging Fc region-modified anti-CD154mAbs successfully prevent the thrombotic complications seen previously (8, 9). Although anti-CD154 agents may be preferable, anti-CD40mAbs have already been applied in phase II clinical trials of human kidney *allo*transplantation (10).

In this review, we consider (i) the optimal age of the islet-source pig, (ii) the potential of genetic modification of the pig, (iii) the selection of the immunosuppressive regimen for pig-to-primate islet xenotransplantation, and (iv) potential steps to reduce the instant blood-mediated inflammatory reaction (IBMIR). We also briefly discuss the possible directions for future research.

## Donor Age

Based on previous studies of pig-to-NHP islet xenotransplantation, pigs can be divided into three age groups: adult (>12 weeks), neonatal (~first 14 days after birth), and fetal. Their characteristics are summarized in **Table 1**. As fetal pig islets are not currently considered ideal sources for xenotransplantation due to defects in β-cell yield and immunogenicity, we will focus on adult and neonatal pigs.

**Table 1:** Characteristics of islets in pigs of different ages.

Characteristic	Fetal	Neonatal	Adult
Isolation procedure	Very simple (no purification)	Simple (No purification)	Difficult
Culture procedure	Resistance to hypoxia and inflammation	Resistance to hypoxia and inflammation	Difficult (Fragile), but not necessary
Early islet loss from IBMIR	Low (inflammation resistance)	Low (inflammation resistance)	Moderate (susceptible to inflammation)
Proliferation *in vivo*	Good	Good	Little
*In vivo* insulin production	Delay >2 months	Delay > 1 month	No delay
*In vitro* GSIS	Poor	Good	Good
Gal expression	High	High	Low
Islet yield (IEQs/pancrease)	~8, 000	25,000-64,000	200,000-720,000
Islet yield (IEQs/g)	NA	5,000-12,500	1,000-16,000
β-Cells % (after culture)	~10%	~25%	~70%
Risk of pathogen transmission	Extremely low	Low	Low
Islet isolation cost	NA	$0.02/IEQ	$0.09/IEQ
Cost	Low	Low	High

Gal, galactose-α1,3-galactose; GSIS, Glucose-stimulated insulin secretion; IBMIR, the instant blood-mediated inflammatory reaction; NA, not available.

### Adult Pig Islets

To date, adult pig islets transplanted into NHPs have displayed the longest survival time (965 days) and have always been considered the primary source for islet xenotransplantation due to their superior islet yield, immediate insulin response, lower Gal expression, and higher β-cell percentage compared with neonatal pigs (**Table 1**). Female adult pigs that have produced >2 litters (retired breeders, usually >2 years old and > 200 kg) are preferred over young adult pigs because they consistently provide a higher yield of high-quality islets (3, 11). We add the ref: Bottino R, 2007 Our previous review summarized the above advantages (3). Using GMP-grade collagenase (collagenase AF-1 and liberase MTF C/T), one adult pig can yield up to 720,000IEQ (12), which is enough for islet xenotransplantation in a diabetic patient of approximately 60kg in weight. However, the limitations of adult pig islets include difficulty in isolation, higher costs for pig maintenance and islet isolation, and poor proliferative capacity (3) (**Table 1**).

### Neonatal Islet Cell Clusters (NICC)

There have been only a few reports using NICC for transplantation into NHPs, with the longest survival being 260 days (13–16). The advantages of NICCs include (I) the need for only a short period of pig maintenance after birth, thus reducing the costs, (ii) easier isolation, thus increasing success and reducing isolation cost ($0.02/IEQ) compared to adult pig islets ($0.09/IEQ) (17), and (iii) greater proliferative capacity (3). However, there are some limitations. First, NICCs must be cultured to reaggregate the islet cluster before transplantation, although various modified culture media, the addition of growth factors, and coculture with mesenchymal stromal cells facilitate NICC islet maturation and function (18–21). Second, there is a delay in the *in vivo* response to glucose after transplantation (that may be >4 weeks in NHPs), and so measuring islet loss is difficult (3).

The difference in the glucose-stimulated insulin secretion index between adult pig islets and NICC remains controversial. Some research has indicated that NICC has a significantly higher stimulation index (4.7± 0.58) than adult pig islets (1.75 ± 0.60) (17), but other studies show the opposite (summarized in **Table 2**) (12, 17, 21–23). Therefore, the glucose-stimulated insulin secretion of adult pig islets and NICC may be equivalent.

**Table 2:** *In vitro* stimulation index of neonatal and adult pig islets.

Reference	Neonatal	Adult	Digestion Enzyme
Vanderschelden et al. (17)	4.7 + 0.58	1.75 + 0.60	Sigma Type V Collagenase Liberase HI
Smith et al. (22)	1.8 ± 0.3	8.5 ± 1.2	
Emamaullee et al. (23)	1.78 ± 0.14	NA	Collagenase
Hassouna et al. (21)	1.7 ± 0.2	NA	Collagenase
Kwak et al. (12)	NA	2.07 ± 0.02	Collagenase P
Kwak et al. (12)	NA	4.73 ± 0.23	Collagenase AF-1*
Kwak et al. (12)	NA	3.87 ± 0.12	Liberase MTF C/T*

*GMP grade; NA, not available.

In summary, a consensus on the optimal age for pig islet xenotransplantation has not been reached. Adult pig islets should be the primary option as better results have been achieved following transplantation into NHPs, but NICCs are regarded as a promising alternative islet source with several significant superiorities.

## Gene Modification

The development of CRISPR/Cas9, an efficient genome editing technique, provides the capacity to produce pigs with multiple genetic modifications for xenotransplantation (**Table 3**) (24–41). We will here mainly focus on gene modification targets for carbohydrate xenoantigens and cellular immune response-related genes.

**Table 3:** Selected gene modifications in pigs of relevance to pig-to-NHP islet xenotransplantation.

Purpose	Modified genes	Ref
Deletion of carbohydrate xeno-antigens	α1,3-galactosyltransferase gene knockout (GTKO)	(24, 25)
	Cytidine monophospho-N-acetylneuraminic acid hydroxylase gene knockout (CMAHKO)	(26)
	β-1,4-N-acetylgalactosaminyltransferase-2 gene knockout (β4GalNT2)	(27)
Prevention of inflammation	Human hemagglutinin-tagged-human hemeoxygenase-1 gene knock-in (HO-1)	(28)
	Soluble human tumor necrosis factor receptor I IgG1-Fc gene knock-in (shTNFRI-Fc)	(28)
Prevention of complement-mediated injury	CD46 gene knock-in	(29)
	CD55 gene knock-in	(30)
	CD59 gene knock-in	(31)
Prevention of coagulation dysfunction	Human thrombomodulin gene knock-in (hTBM)	(32)
	Human endothelial protein C receptor gene knock-in (EPCR)	(33)
	Human tissue factor pathway inhibitor-2 knock-in (hTFPI)	(34)
	CD39 gene knock-in	(35)
Protection against cellular immune response	Cytotoxic T-lymphocyte antigen-4 immunoglobulin (CTLA4-Ig) or LEA29Y transgene (CTLA4-Ig mutation)	(36)
	MHC class II transactivator knockdown (CIITA-DN)	(37)
	β2-microglobulin knock-out (B2MKO)	(38)
	CD47 gene knock-in	(39)
	Programmed cell death ligand 1 gene knock-in (PD-L1)	(40)

### Carbohydrate Xenoantigen Genes

A consensus has been reached that the three known carbohydrate xenoantigen genes (Gal, Neu5Gc, Sda) should be knocked-out for pig-to-human organ transplantation (**Table 4**), but this is not ideal for pig-to-NHP organ transplantation because of the problems associated with the ‘4th xenoantigen’ (discussed in 42–46). It is well-known that pig organ grafts from CMAHKO pigs are associated with increased NHP IgM and IgG binding and serum complement-mediated cytotoxicity, resulting in acute xenograft rejection (42–46).

**Table 4:** Known carbohydrate xenoantigens expressed on pig cells.

Carbohydrate (Abbreviation)	Responsible enzyme	Gene-knockout pig
1.Galactose-α1,3-galactose (Gal)	α1,3-galactosyltransferase	GTKO
2.N-glycolylneuraminic acid (Neu5Gc)	CMAH	CMAH-KO
3.Sd^a^	β-1,4N-acetylgalactosaminyltransferase.	β4GalNT2-KO

CMAH, Cytidine monophosphate-N-acetylneuraminic acid hydroxylase (CMAH).

To our knowledge, the transplantation of TKO pig islets into NHPs has not been reported, and it remains unknown whether the ‘4^th^ xenoantigen’ is exposed in TKO pig islets as it is in vascular endothelial cells. Whether TKO islets would provide an advantage in this regard remains uncertain.

Of relevance to this point, there were no statistically significant differences in human IgM and IgG binding to isolated islet cells from GTKO/hCD46 and GTKO/hCD46/NeuGcKO pigs (47). Knockout of CMAH may therefore possibly have a different effect in islets than in solid organs. In one report, GTKO/CMAHKO pigs developed pathological features that are similar to those seen in anemia, possibly associated with variations in glycosylation on the red blood cell membranes of these pigs (48). Obukhova et al. have reviewed CMAH comprehensively (49).

If neonatal pigs are the source of islets (i.e., NICCs), in which expression of Gal is considerable, the deletion of expression of Gal (and possibly of Neu5Gc and Sda) will be advantageous.

Differences in N- and O-glycan profiles between human and porcine islets might prove to be the next gene modification sites. Novel xenoantigens include complex-type N-glycans with terminal neuraminic acid residues and high-mannose-type N-glycans with core fucosylation (50). Carbohydrate antigen microarrays in pigs and cynomolgus monkeys have revealed natural non-αGal antigens (e.g., Tn antigen, T antigen, GM2 glycolipid) and novel carbohydrate structures (e.g., Galβ1-4GlcNAcβ1-3Galβ1 and N-linked glycans with Manα1-6 (GlcNAcβ1-2Manα1-3)Manβ1-4GlcNAcβ) that are responsible for the IgM and IgG anti-carbohydrate antibody responses (51, 52). These findings suggest future gene modification sites to eliminate anti-carbohydrate antibody responses in pig-to-primate islet xenotransplantation.

For future studies of the 4th xenoantigen(s), several sources might be helpful, e.g., the database of Glycomics (http://www.functionalglycomics.org/). The National Center for Functional Glycomics (NCFG) (https://ncfg.hms.harvard.edu/) offers a CFG mammalian-type glycan microarray, with 600 glycans present, that might be helpful in studying xenoantigens in the future.

### Cellular Immune Response-Related Genes

Progress in gene modification aimed at protecting xenografts from the adaptive immune response has been made recently. For example, knock-in of CTLA4-Ig or the high-affinity variant LEA29Y (36, 53), knockout or knockdown swine leukocyte antigen (SLA) class I and class II (37, 54), and *in vitro* tests on SLA class I and class II-silenced cells have reported significantly reduced xenogeneic T cell and natural killer cell responses, and antibody-mediated cell-dependent responses to islet cell clusters (55). However, CTLA4-Ig or LEA29Y transgenic pigs face the problems of hypoimmunity (36, 56).

Immune checkpoint blockade is a promising approach to control pathogenic immune responses. Immunomodulation with PD-L1 improves islet allotransplantation outcomes (57–63), and may facilitate successful xenotransplantation. PD-L1 is a ligand that reduces the proliferation and activation of T cells, B cells, and monocytes through interaction with PD-L1 receptors on these cells, and prevents cell-mediated lysis from CD8^+^ T cells by reducing their proliferation and cytokine secretion (40). Programmed cell death protein 1 blockade has successfully achieved clinical objectives in the treatment of cancer (64–66). In xenotransplantation, pigs transgenic for PD-L1 have been successfully generated, and cells from these pigs prevent human T cell cytotoxicity and B cell activation *in vitro* (57, 58), with similar results in a pig-to-rat xenotransplantation model (67). In contrast, islet PD-L1 deficiency has been associated with increased allograft rejection and increased inflammatory cell infiltration (68). Testing of the transplantation of pig islets expressing PD-L1 in NHPs should be a future research direction.

In summary, whether the 4th xenoantigen is exposed in islets after CMAHKO remains uncertain, and more research on the cellular response (that will be the next obstacle to explore) is required (69).

## Immunosuppressive Regimen

The main objective of the immunosuppressive regimen is to inhibit T cell activation and prevent subsequent T cell-dependent dendritic cell activation and activation of B cells and macrophages. Immunosuppressive regimens based on conventional (FDA-approved) therapy have proved inadequate, although islet graft survival of 222 days has been reported (70). In contrast, blockade of the CD40/CD154 costimulation pathway has resulted in maximal islet graft survival of 965 days **(** **Table 5**) (7, 13, 70–76). The major mechanistic effects, advantages, and side-effects of the key immunosuppressive agents of relevance to xenotransplantation have been reviewed by Bikhet and his colleagues (77). Samy et al. have reviewed the role of costimulation pathway blockade in xenotransplantation (78). Here we will focus on novel immunosuppressive regimens based on blockade of the CD40/CD154 costimulation pathway.

**Table 5:** Immunosuppressive protocols associated with prolonged periods of insulin-independence and islet graft survival.

Major agent	Islet-source pig	Immunomodulatory regimen	Maximum Insulin independence	Maximum graft survival	Ref
Anti-CD154	WT (adult)	Anti-ICAM-1 mAbs (MD-3), anti-CD154 mAbs (5C8), Sirolimus, TNF-a-neutralizing mAb (adalimumab), Anakinra, Ganciclovir, Clopidogrel, Heparin	520d	520d	(71)
	WT (adult)	ATG, anti-CD154 mAbs (5C8), Sirolimus, CVF, TNF-a-neutralizing mAb (adalimumab)	603d	603d	(72)
	hCD46 (adult)	ATG, Anti-CD154 mAbs (ABI7953), MMF, Dextran sulfate, Prostacyclin, Methylprednisolone, Aspirin, Ganciclovir, Famotidine, Heparin	365d	365d	(73)
	GTKO, hCD46, hCD39, hTFPI (adult)	ATG, Anti-CD154 mAbs (h5c8), MMF, Dextran sulfate, Prostacyclin, Methylprednisolone, Aspirin, Ganciclovir, Famotidine, Heparin	365d	365d	(73)
	hCD46 (adult)	ATG, Anti-CD154 mAb (ABI7953), Dextran sulfate, Methylprednisolone, Aspirin, Prostacyclin	396d	396d	(74)
Anti-CD40	WT (neonatal)	Anti-CD40 mAbs (Chi220), aIL-2R (Basiliximab), Belatacept, Sirolimus	203d	>203d	(13)
	WT (adult)	Anti-CD40 mAbs (2C10R4), Sirolimus, ATG, CVF, Tacrolimus, Adalimumab, Methylprednisolone	266d	320d	(75)
Anti-CD154 plus Anti-CD40	WT (adult)	ATG, CVF, anti-CD154 mAbs (5C8), Anti-CD40 mAbs (2C10R4), Sirolimus, TNF-a-neutralizing mAb (adalimumab), Treg	965d	965d	(7)
Conventional	WT (adult)	ATG, Rituximab, Belimumab, Sirolimus, Tacrolimus, Tofacitinib, Adalimumab, Anakinra, CVF, IVIg	130d	201d	(76)
	WT (adult)	ATG, Belimumab, Sirolimus, Tacrolimus, Abatacept, Tofacitinib, Adalimumab, Anakinra, Tocilizumab, IVIg, Aspirin	90d	222d	(70)

aIL-2R, IL-2 receptor-specific antibody; ATG, anti-thymocyte globulin; CVF, cobra venom factor; GTKO, 1,3-galactosyltransferase gene-knockout; hTFPI, knock-in human tissue factor pathway inhibitor-2; ICAM-1, intercellular cell adhesion molecule-1; IVIg, intravenous immunoglobulin; mAbs, monoclonal antibodies; MMF, mycophenolate mofetil; Treg, regulatory T cell; WT, wild type.

### Immunosuppressive Regimens Based on Anti-CD40mAbs

Anti-CD40mAbs are a chimeric form of Fab combined with IgG Fc fragments to prevent the stimulation of B and T cells through blockade of the CD40/CD154 pathway, which also participates in regulating thrombosis, tissue inflammation, and hematopoiesis (79). Unlike anti-CD154mAbs, no significant thrombogenic complications have been observed in anti-CD40mAb studies (10). Islet graft survivals are summarized in **Table 5**.

To date, some anti-CD40mAbs have completed phase II clinical trials of allotransplantation (but *not* in islet transplantation). These included bleselumab (ASKP1240), iscalimab (CFZ533), and BI 655064 (10, 80, 81). Among them, ASKP1240 demonstrated good results with a favorable benefit-risk ratio and no thromboembolic events in a phase II clinical kidney transplantation trial (10). Treatment with 2C10R4 was associated with the longest pig islet graft survival in NHPs to date (maximum insulin-independence 950 days, maximum graft survival 965 days) (7). However, anti-CD40mAbs may be associated with adverse effects, e.g., a temporary increase in liver enzymes (ASKP1240) (82, 83), significant depletion of peripheral blood B cells (Chi220) (13), and inhibition of T regulatory cell (Treg) expansion (2C10R4) (84).

### Immunosuppressive Regimens Based on Anti-CD154 Agents

Anti-CD154 agents also provide efficient CD40/CD154 pathway blockade (85), but were originally associated with thromboembolic complications (BG9588, hu5c8, IDEC-131, ABI793) (86–88), although the situation with IDEC-131 remains controversial (89). They were demonstrated to be preferable to anti-C40mAbs in pig islet transplantation in NHPs (**Table 5**) (72, 75). Modifications to the Fc region on CD154 agents, the binding site for the Fc receptor (FcgRIIA) on platelets (85), appear to have eliminated thromboembolic events (e.g., CDP7657 and BMS-986004 in rhesus macaques, and MEDI4920 in cynomolgus monkeys) (77). To date, CDP7657, BMS-986004, and MEDI4920 have completed phase I or II clinical trials (*not* in islet transplantation) without obvious complications (8, 9, 90–92).

Overall, although anti-CD40mAbs have proved successful in pig-to-NHP islet xenotransplantation, the new anti-CD154 agents may prove preferable for clinical trials (**Table 6**) (9, 10, 75, 80–83, 90, 92–102). Of importance, ongoing studies at the Massachusetts General Hospital indicate that *monotherapy* with an anti-CD154mAb (with *no* additional immunosuppressive therapy) prevents rejection of heterotopic heart and life-supporting kidney *allo*grafts in monkeys (Robin Pierson and Tatsuo Kawai, personal communications). This regimen, or a modification of it, has not yet been tested in xenograft models

**Table 6:** Agents that block the CD40/CD154 costimulation pathway that are currently in clinical trials and preclinical studies, an update of Bikhet 2021 (58).

Drug and company	Clinical trials	Results
**Anti-CD40**		
Bleselumab	Phase Ia/Ib:	
(ASKP124/4D11)	NCT01279538 (60, 72)	Well-tolerated in healthy humans and in kidney transplant recipients
Astellas	Phase II:	
	NCT01780844 (9)	well tolerated in kidney transplant recipients
	NCT01585233 (78)	well tolerated in moderate-to-severe plaque psoriasis patients
	NCT02921789	Kidney transplantation (without results)
Iscalimab	Phase I:	
(CFZ533)	NCT02089087 (73)	well tolerated in Rheumatoid Arthritis
Novartis	Phase I/II:	
	NCT02217410 (74, 75)	well tolerated in kidney transplant recipients
	Phase II:	
	NCT02291029 (76)	Has therapeutic potential in primary Sjogren’s syndrome patients
	NCT02713256 (58)	Has therapeutic potential in Graves’ disease patients
	NCT02565576	Has therapeutic potential in Severe Myasthenia Gravis
	NCT03663335	Kidney transplantation (without results)
	NCT03781414	Liver transplantation (without results)
	NCT03610516	Lupus nephritis (without results)
	NCT03905525	Sjogren’s syndrome (without results)
	NCT04129528	Type 1 Diabetes (without results)
	NCT03656562	SLE (without results)
BI 655064	Phase I:	
Boehringer Ingelheim	NCT01751776 (77)	Well-tolerated in healthy humans
	Phase II:	
	NCT01751776 (59)	Safety in rheumatoid arthritis patients with inadequate response to methotrexate
	NCT03385564	Lupus nephritis (without results)
	NCT02770170 (78)	Lupus nephritis (did not meet its primary CRR endpoint)
KPL-404	Phase I:	
Kiniksa	NCT04497662 (79)	Well-tolerated in healthy humans
2C10R4	Preclinical study (55, 80, 81)	Prolonged graft survival in pig-to-NHP cardiac and islet xenotransplantation, NHP islet allotransplantation
NIH NHP Resource Center		
**Anti-CD154**		
Dapirolizumab	Phase I:	
(CDP7657)	NCT01093911 (69)	Well tolerated in healthy humans and in patients with SLE
UCB AND BIOGEN	NCT01764594 (8)	Safety and efficacy in SLE patients
	NCT04571424	Healthy human (without results)
	Phase II:	
	NCT02804763 (71)	Well tolerated in healthy human and SLE. Has therapeutic potential in SLE
	Phase III:	
	NCT04294667	SLE (without results)
	NCT04976322	SLE (without results)
Letolizumab	Phase I/II:	
(BMS-986004)	NCT02273960	Safety in Immune thrombocytopenic purpura (ITP)
BMS	Phase I/II:	
	NCT03605927	Graft-versus-host disease (GVHD) (without results)
VIB4920	Phase I:	
(MEDI4920)	NCT02780388	Well tolerated in patients with rheumatoid arthritis
VielaBio	NCT02151110	Well tolerated in healthy adults
	Phase II:	
	NCT04046549	Kidney transplantation (without results)
	NCT04129164	Sjogren’s syndrome (without results)
	NCT04163991	Rheumatoid arthritis (without results)
	NCT04174677	Kidney Transplantation (without results)

GVHD, graft-vs-host disease; ITP. immune thrombocytopenic purpura; mAb, monoclobal antibody; PEG, polyethylene glycol; SLE, systemic lupus erythematosus; TCP, thrombocytopenic purpura; NA, not available.

Bikhet et al. published an immunosuppressive regimen that has proved moderately successful in pig solid organ transplantation in NHPs (77), but such a regimen may be too intensive to warrant use in patients with islet xenografts.

## The Instant Blood-Mediated Inflammatory Reaction (IBMIR)

After infusion of islets into the portal vein (the preferred site at present), a substantial percentage of islets are lost in the immediate post-transplant period through an inflammatory response termed IBMIR. The loss is significantly greater if the islets are xeno-islets, e.g., pig islets into NHPs and pig islets to human blood *in vitro* (103–107). Coagulation, platelet aggregation, complement activation, and neutrophil and monocyte infiltration play roles in this reaction (108). Several approaches to reduce the loss of islets have been explored, e.g., anticoagulation, complement depletion (109), and modified islet culture medium (110), but none has been entirely successful yet. The transplantation of islets from pigs with one or multiple genetic modifications may help protect the islets from early injury and loss (14, 74, 111–115). Moreover, alternative transplantation sites in intrapleural space greatly reduced IBMIR (116).

It is beneficial to add heparin or dextran sulfate to the peri-transplant regimen for their anticoagulant and complement-modulating properties that reduce islet loss from IBMIR (109, 117–120). Low molecular dextran sulfate at low doses demonstrated good results in the prevention of IBMIR in phase II clinical islet *allo*transplantation study (NCT00789308) (119). Nanoparticle-based techniques improve the therapeutic efficacy of heparin. For example, polymeric nanocoating islets with heparin-polyethylene glycol (PEG) or chondroitin sulfate-PEG in an NHP islet allotransplant model was associated with significantly longer islet survival with reduced loss to IBMIR compared with PEG and naked islets (121, 122). Conjugated nanoparticles (heparin-immobilized superparamagnetic iron oxide) conjugated onto the surface of the islets attenuated IBMIR in a rat-to-mouse islet xenotransplantation model (123). Islet-surface modifications with streptavidin-CD47 protein, a chimeric construct expressing CD47 on the extracellular domain, efficiently prevent islet loss from IBMIR (124).

Cibinetide (Araim Pharmaceuticals Inc., Tarrytown, NY, USA) (a non-hematopoietic erythropoietin analogue) also showed islet-protective effects by reducing IBMIR-induced platelet consumption (125). Based on these studies, agents that reduce IBMIR, combined with the transplantation of islets from genetically-engineered pigs (e.g., pigs not expressing the known carbohydrate xenoantigens, but expressing human complement- and coagulation-regulatory proteins), and an optimal immunosuppressive regimen may increase graft survival and the therapeutic efficacy of islet xenotransplantation.

## Comment

Key factors in successfully developing pig islet xenotransplantation include determination of the optimal age of the islet-source pig (adult or neonatal), the optimal genetic modifications that should be made to the pig, and the optimal immunosuppressive regimen that should be administered to the recipient. Whether the ‘4th’ xenoantigen is problematic in the pig-to-NHP islet transplantation model needs to be clarified. More attention needs to be directed to genetic modifications that might reduce the instant blood-mediated inflammatory reaction and/or the adaptive immune response to pig islets. The advantages and disadvantages of immunosuppressive regimens based on anti-CD40 and anti-CD154 agents require clarification. Since the first case of successful pig-to-human kidney and heart transplantation had been reported recently (126, 127), we anticipate that pig islet xenotransplantation will become clinically successful when these remaining questions have been resolved.

## Author Contributions

LM, DC, and ZP initiated the review. LM and GS wrote the manuscript. DC, YL, JC, SZ, JD, YH, YN, YZ, and ZC revised the manuscript. All authors contributed to the article and approved the submitted version.

## Funding

This work was supported by grants from the Shenzhen Foundation of Science and Technology (grant numbers GJHZ20200731095207021), the National Key R&D Program of China (2017YFC1103704) and from the Special Funds for the Construction of High Level Hospitals in Guangdong Province (2019).

## Conflict of Interest

The authors declare that the research was conducted in the absence of any commercial or financial relationships that could be construed as a potential conflict of interest.

## Publisher’s Note

All claims expressed in this article are solely those of the authors and do not necessarily represent those of their affiliated organizations, or those of the publisher, the editors and the reviewers. Any product that may be evaluated in this article, or claim that may be made by its manufacturer, is not guaranteed or endorsed by the publisher.

## Abbreviations

CMAH, cytidine monophospho-N-acetylneuraminic acid hydroxylase; Gal, galactose-α1,3-galactose; GTKO, 1,3-galactosyltransferase gene-knockout; IBMIR, instant blood-mediated inflammatory reaction; mAbs, monoclonal antibodies; Neu5Gc, N-glycolylneuraminic acid; NHP, nonhuman primate; NICC, neonatal islet cell clusters; PD-L1, programmed cell death ligand 1.
